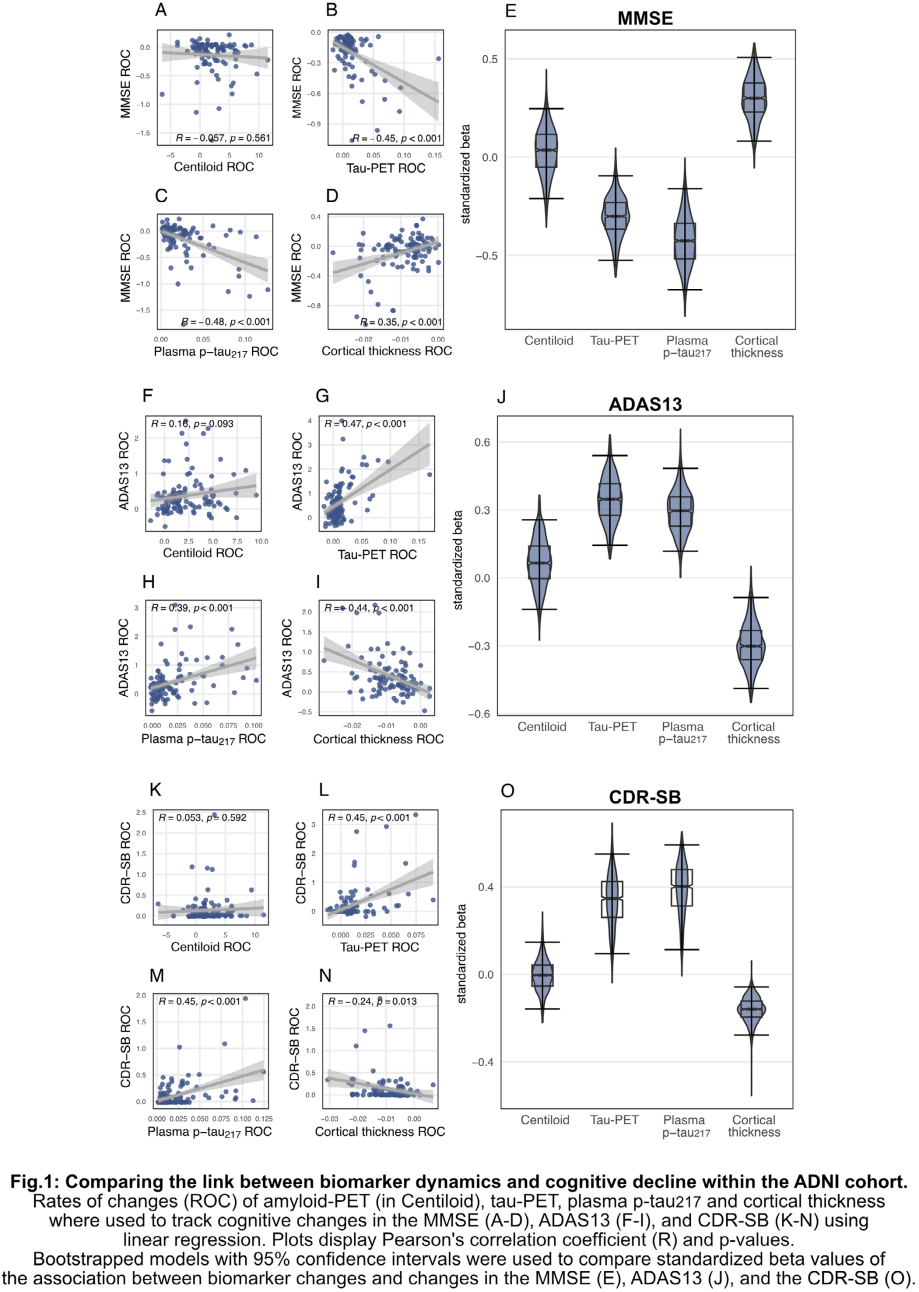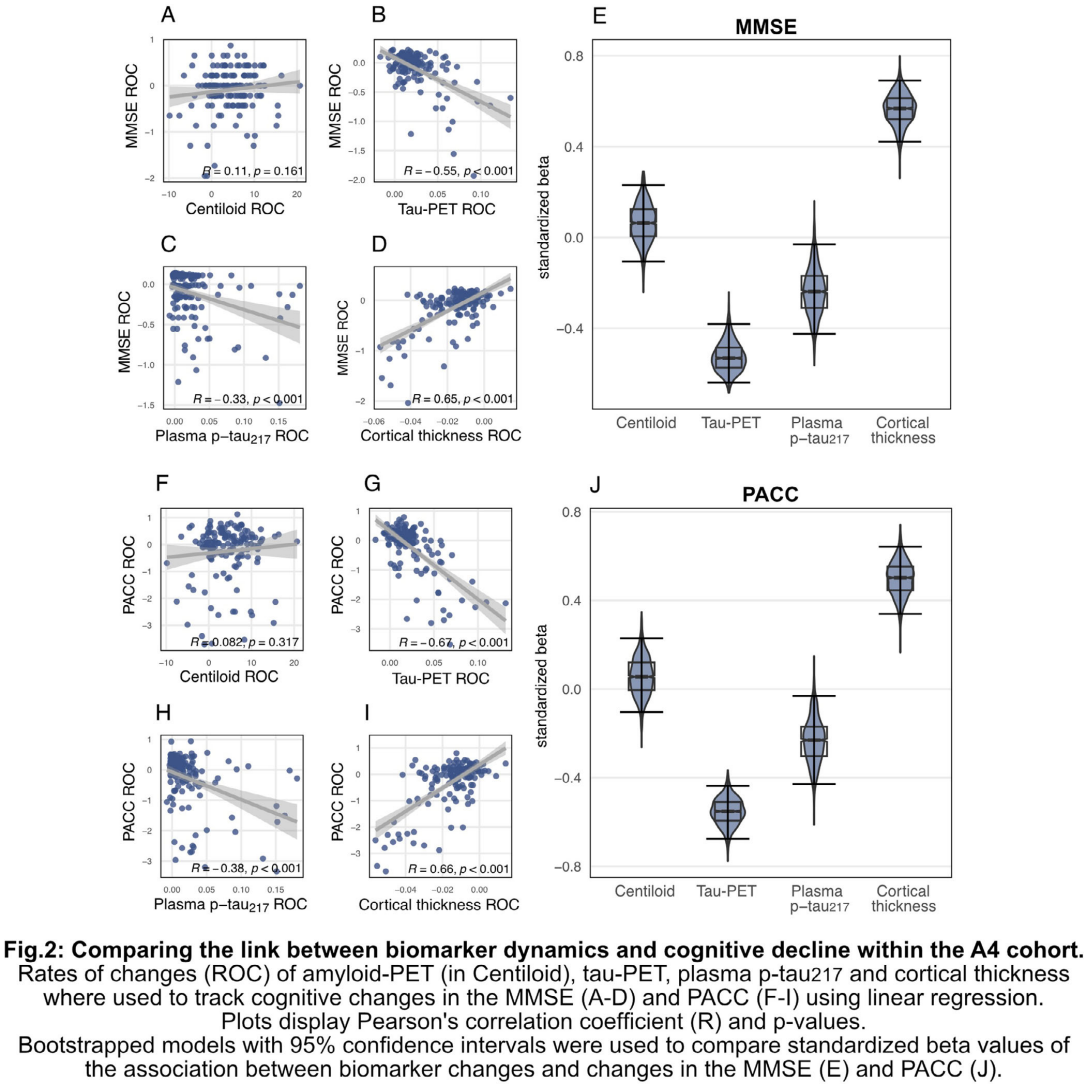# Plasma *p*‐tau_217_ as a suitable biomarker for monitoring cognitive changes in Alzheimer’s disease

**DOI:** 10.1002/alz70862_110180

**Published:** 2025-12-23

**Authors:** Davina Biel, Anna Steward, Anna Dewenter, Amir Dehsarvi, Zeyu Zhu, Sebastian Roemer‐Cassiano, Lukas Frontzkowski, Fabian Hirsch, Matthias Brendel, Nicolai Franzmeier

**Affiliations:** ^1^ Institute for Stroke and Dementia Research (ISD), University Hospital, LMU Munich, Munich, Bavaria Germany; ^2^ Department of Nuclear Medicine, University Hospital, LMU Munich, Munich, Bavaria Germany; ^3^ German Center for Neurodegenerative Diseases (DZNE), Munich Germany; ^4^ Munich Cluster for Systems Neurology (SyNergy), Munich, Bavaria Germany; ^5^ University of Gothenburg, The Sahlgrenska Academy, Institute of Neuroscience and Physiology, Psychiatry and Neurochemistry, Gothenburg Sweden; ^6^ Institute for Stroke and Dementia Research (ISD), LMU University Hospital, LMU, Munich, Bavaria Germany

## Abstract

**Background:**

With the approval of anti‐amyloid therapies in Alzheimer’s disease (AD), surrogate biomarkers are urgently needed to monitor treatment effects that translate into clinical benefits. Candidate biomarkers, including amyloid‐PET, tau‐PET, plasma phosphorylated tau (*p*‐tau), and MRI‐assessed atrophy, capture core pathophysiological changes in AD. While cross‐sectional biomarker assessments are critical for diagnosis and staging, biomarker change rates may better reflect disease dynamics, making them more suitable for monitoring treatment efficacy. Therefore, we determined which biomarker most effectively tracks cognitive changes in AD, identifying those best suited for efficient monitoring of disease‐modifying treatments.

**Method:**

We leveraged ADNI (*N* = 108) and A4 (*N* = 151) participants with longitudinal AD biomarker data (global amyloid‐PET, temporal meta tau‐PET, plasma *p*‐tau_217_, MRI‐assessed cortical thickness in the AD signature region) together with cognitive assessments (ADNI: MMSE, ADAS13, CDR‐SB; A4: MMSE, PACC). Linear mixed models were used to calculate change rates for biomarkers and cognition. To test whether biomarker changes track cognitive decline, linear models were applied, to test biomarker change rates as a predictor of cognitive change rates. Standardized beta values from bootstrapped linear models were extracted to compare the strengths of correlations between biomarkers and cognitive decline. For non‐parametric comparisons, 95% confidence intervals (CIs) of standardized beta values were compared. Models were controlled for age, sex, education, and baseline cognition, with ADNI models additionally adjusted for clinical status.

**Result:**

In both cohorts, changes in temporal tau‐PET, plasma *p*‐tau_217_, and MRI‐assessed cortical thickness were associated with cognitive decline (ADNI: Figure 1; A4: Figure 2). Amyloid‐PET changes showed no significant association with cognitive changes (ADNI: Figure 1A+F+K; A4: Figure 2A+F). Bootstrapping confirmed that tau‐PET, plasma *p*‐tau_217_, and cortical thickness track cognitive decline, but not amyloid‐PET (ADNI: Figure 1E+J+O; A4: Figure 2E+J). Overlapping CIs for tau‐PET and plasma *p*‐tau_217_ indicated comparable predictive accuracy.

**Conclusion:**

Our findings demonstrate that tau‐PET and plasma *p*‐tau_217_ are robust biomarkers for monitoring cognitive changes, with plasma *p*‐tau_217_ offering a cost‐effective, scalable alternative for clinical use. Changes in amyloid‐PET do not reliably reflect cognitive decline, limiting its utility as a treatment monitoring tool. Although cortical thickness correlates with cognitive changes, its application is limited by pseudoatrophy and volume loss induced by anti‐amyloid antibody treatments.